# Hepatocellular Carcinoma: Updates in Pathogenesis, Detection and Treatment

**DOI:** 10.3390/cancers12102729

**Published:** 2020-09-23

**Authors:** Maria L. Martínez-Chantar, Matias A. Avila, Shelly C. Lu

**Affiliations:** 1 CIC bioGUNE, Bizkaia Technology Park, 48160 Derio, Spain; 2 National Institute for the Study of Liver and Gastrointestinal Diseases (CIBERehd, Carlos III Health Institute), 28029 Madrid, Spain; 3 Program of Hepatology, Center for Applied Medical Research (CIMA), University of Navarra, 31008 Pamplona, Spain; 4 Karsh Division of Gastroenterology and Hepatology, Cedars-Sinai Medical Center, Los Angeles, CA 90048, USA

**Keywords:** hepatocellular carcinoma, risk factors, molecular mechanisms, liquid biopsy, early diagnosis, multikinase inhibitors, immune checkpoint inhibitors

Hepatocellular carcinoma (HCC) is the most frequent primary liver cancer and the second most common cause of cancer mortality worldwide [1]. The prognosis of HCC patients is very poor. The rates of HCC incidence and mortality are almost equivalent [2] and have increased across most countries over the past three decades [3]. HCC development is closely associated with the presence of chronic liver disease and cirrhosis, albeit the risk factors underlying this condition vary geographically. Hepatitis B virus (HBV) infection and aflatoxin B1 exposure are predominant risk factors in Asia and Africa, while hepatitis C virus (HCV) infection and alcohol consumption are the main risk factors in Europe, the USA and Japan [3,4,5]. Non-alcoholic fatty liver disease (NAFLD) is currently the most prevalent liver disease worldwide, and approximately 60% of biopsied NAFLD patients have non-alcoholic steatohepatitis (NASH) [3]. Importantly, patients with NASH are at high risk of developing HCC even without presenting established cirrhosis [6]. With widespread HBV vaccination and the advent of direct-acting antiviral drugs for HCV infection, NAFLD and associated conditions such as diabetes and obesity are emerging as major global risk factors for HCC. In view of the dismal prognosis of HCC patients, implementing preventive strategies would be an ideal approach to quell the incidence of the disease. Obvious interventions include advocating HBV vaccination in endemic regions, achieving HCV eradication with direct-acting antivirals, promoting healthy nutrition and weight reduction, improving diabetes control, and avoiding excessive alcohol consumption. Still, the implementation of these measures is not always feasible. Chemopreventive strategies have been proposed. However, the identification of chemoprevention targets is not straightforward, and the design and conduction of clinical trials for chemoprevention is resource intensive [3]. HCC screening/surveillance is also essential for early disease detection and the application of effective treatments that reduce disease-associated mortality. For patients with cirrhosis, or with hepatitis B infection without cirrhosis, ultrasonography combined with serum alpha-fetoprotein levels is the currently recommended approach for widespread screening, although computed tomography (CT) and magnetic resonance imaging (MRI) may offer higher sensitivity for small lesions [3,4]. For patients with NAFLD, ultrasonography has decreased diagnostic accuracy, and in these cases, testing for the presence of genetic variants (e.g., I148M of *PNPLA3*) may inform on HCC risk [7]. The performance of histological biomarkers in combination with clinical data has shown promise for surveillance and early diagnosis [4]. Besides established histoprognostic factors (i.e., tumor differentiation and vascular invasion) the identification of gene signatures with prognostic value has been an area of intense research over the past couple of decades. Nevertheless, mandatory biopsy is not advocated in clinical guidelines, which, together with sampling variability and tumor molecular heterogeneity, have compromised the translation of gene signature assessment into clinical practice [8]. To circumvent these issues, strong efforts are currently being made in the field of liquid biopsy. In this approach, tumor components, including tumor cells, tumor DNA or tumor-derived extracellular vesicles, are analyzed in circulation. It is expected that liquid biopsy may help in early HCC detection, disease prognosis, and treatment response [9,10,11].

From a therapeutic perspective, surgical therapies can be applied in patients with single lesions at an early stage, with well-preserved liver function. However, as many as 70% of these patients develop tumor recurrence after five years [4,8,10]. Therefore, the identification of systemic therapies that may prevent this recurrence, either true recurrence due to dissemination or late recurrence due to de novo tumor formation, is of paramount importance. Locoregional therapies, such as radiofrequency ablation and transarterial therapies, are considered for patients that are not candidates for surgery. Transarterial chemoembolization (TACE), in which cytotoxic agents are injected through tumor-feeding vessels, and selective internal radiation therapy (SIRT) in which microspheres loaded with yttrium-90 are administered, have shown clinical efficacy [4,10]. However, the combination of TACE or SIRT with anti-angiogenic systemic therapies has shown no clinical benefit [8].

When considering the treatment of HCC at more advanced stages, it was clear early on that this tumor is highly resistant to conventional systemic anti-cancer therapies [12]. This realization stimulated the search for more tailored therapies targeting pathways and mechanisms essential for tumor cell survival [12]. Early molecular and cellular studies pointed to interference with growth factor signaling pathways as potential points of pharmacological intervention in anti-HCC therapy [12]. After several failed trials with mono-specific targeted agents, such as epidermal growth factor receptor inhibitors, this approach led to the identification of multikinase inhibitors such as sorafenib, which in carefully designed clinical trials demonstrated efficacy in patients with advanced disease [13]. Sorafenib was followed by other related drugs, such as regorafenib and the even more promiscuous tyrosine kinase inhibitor lenvatinib, which showed improved clinical performance [14]. Experimental and clinical studies suggest the idea that the efficacy of these inhibitors is mediated not only through their interaction with tumor cells, but also with the pro-inflammatory, fibrogenic and pro-angiogenic tumor microenvironment [15]. The unraveling of the genetic features of HCC through the application of high-throughput gene expression analyses and next-generation sequencing (NGS) technologies has provided an increasingly precise molecular portrait. The most recurrent mutations, DNA copy number alterations and associated changes in gene expression contributing to hepatocarcinogenesis, have been described in an increasingly comprehensive manner [16,17,18]. These and other studies allowed for the classification of HCCs into molecular subgroups with associated molecular and clinical phenotypes [13,17]. The most frequent (95% of tumors) and earliest genetic alteration in hepatocarcinogenesis is the overexpression of telomerase reverse transcriptase (*TERT*) [18]. Unfortunately, pharmacological targeting of TERT has not been successfully achieved yet [17]. More recently, epigenetic alterations are increasingly being recognized as potential driving factors in HCC development, and mutations in epigenetic modifiers can be found in up to 50% of HCCs [19,20]. Importantly, epigenetic mechanisms can be pharmacologically targeted, and the development of the so-called “epidrugs”, already used to treat hematological malignancies, has opened a new avenue for solid tumor therapy, including that of HCC [20,21].

Cancer immunotherapy has also reached the field of HCC treatment. Recent clinical trials have demonstrated promising activity against HCC of immune checkpoint inhibitor (ICI) antibodies targeting cytotoxic T lymphocyte-associated protein-4 (CTLA-4), or anti-programmed cell death protein-1 (PD-1) and its ligand, programmed cell death ligand-1 (PD-L1) [22,23,24]. In spite of being generally well tolerated, the efficacy of ICI-based monotherapy, such as that of multikinase inhibitors, is still suboptimal, and combinatory strategies including ICI and anti-angiogenic agents are actively being pursued [14,23].

Although current HCC therapy is still far from optimal, the promising advances we are witnessing today undoubtedly come from an increased understanding of the fundamental aspects of hepatocarcinogenesis. Better knowledge of tumor cell metabolism [25], HCC epigenetics [26], HCC cancer stem cells [27] and the immunology of HCC, and the hepatic environment [28] will provide new avenues for improved therapeutic intervention. Moreover, further exploration of the role of the gut microbiome and the gut–liver axis in hepatocarcinogenesis will also deliver potential diagnostic tools and therapeutic targets [29]. In this Special Issue “Hepatocellular Carcinoma: Updates in Pathogenesis, Detection and Treatment” of *Cancers*, we aim to bring together a series of articles providing novel information or insightful reviews of the key aspects of HCC pathogenesis, diagnosis and therapy.
